# 

**Published:** 1879-02

**Authors:** 


CONTENTS.

| | Page |
|---|---|
| The Church and the World | 717 |
| France and America | 721 |
| The Same all the Time | 722 |
| The Great Battle | 723 |
| Living Yet | 724 |
| Interesting Facts | 725 |
| Near-Sighted | 727 |
| Jestings | 728 |
| True Teachings | 733 |
| The Utilities | 733 |
| Smoking | 735 |
| Popular Fallacies | 736 |
| Servant Girls | 743 |

